# Sexual Dimorphism in Hematocrit Response Following Red Blood Cell Transfusion of Critically Ill Surgical Patients

**DOI:** 10.5402/2012/298345

**Published:** 2012-03-22

**Authors:** Fredric M. Pieracci, Carlton C. Barnett, Nicole Townsend, Ernest E. Moore, Jeffery Johnson, Walter Biffl, Denis D. Bensard, Clay C. Burlew, Andrew Gerber, Christopher C. Silliman

**Affiliations:** ^1^Department of Surgery, Denver Health Medical Center, University of Colorado Health Science Center, 777 Bannock Street MC0206, Denver, CO 80206, USA; ^2^Departments of Research and Information Services, Bonfils Blood Center, Denver, CO 80206, USA

## Abstract

The change in hematocrit (ΔHct) following packed red blood cell (pRBCs) transfusion is a clinically relevant measurement of transfusion efficacy that is influenced by post-transfusion hemolysis. Sexual dimorphism has been observed in critical illness and may be related to gender-specific differences in immune response. We investigated the relationship between both donor and recipient gender and ΔHct in an analysis of all pRBCs transfusions in our surgical intensive care unit (2006–2009). The relationship between both donor and recipient gender and ΔHct (% points) was assessed using both univariate and multivariable analysis. A total of 575 units of pRBCs were given to 342 patients; 289 (49.9%) donors were male. By univariate analysis, ΔHct was significantly greater for female as compared to male recipients (3.81% versus 2.82%, resp., *P* < 0.01). No association was observed between donor gender and ΔHct, which was 3.02% following receipt of female blood versus 3.23% following receipt of male blood (*P* = 0.21). By multivariable analysis, recipient gender remained associated significantly with ΔHct (*P* < 0.01). In conclusion, recipient gender is independently associated with ΔHct following pRBCs transfusion. This association does not appear related to either demographic or anthropomorphic factors, raising the possibility of gender-related differences in recipient immune response to transfusion.

## 1. Introduction

Transfusion of critically ill patients with packed red blood cells (pRBCs) is commonplace; nearly all anemic intensive care unit (ICU) patients with a length of stay (LOS) ≥ 7 days receive at least one pRBCs transfusion [1, 2]. Despite the widespread use of pRBCs transfusions as a treatment of ICU anemia, there are a paucity of data demonstrating their efficacy in terms of improvement of oxygen delivery, oxygen consumption, and ultimately patient outcome [3–6]. By contrast, research over the last several decades has elucidated the detrimental effects of blood product transfusion, particularly with respect to immune suppression, infection, inflammation, and organ failure [5, 7–9]. Such phenomena are believed to involve both transfusion and host response components. In the case of the former, proinflammatory components within the transfusion, such as leukocytes, cytokines, and antibodies, inflict damage directly upon recipient organ systems (including the immune system). In the case of the later, the recipient immune system reacts to antigens within the transfused blood, such as proinflammatory cytokines, lipid mediators, and the erythrocytes themselves, which may in turn cause collateral damage to the host [10, 11].

Research on the impact of gender on ICU outcomes has received recent attention and yielded conflicting results. Several studies have reported sexual dimorphism with respect to both overall and infection-related mortality among critically ill patient cohorts ranging from severe trauma [12, 13] to postcardiac arrest [14]. Demonstration of sexual dimorphism in both risk and survival of severe sepsis [15, 16] has led investigators to invoke gender-related differences in immune response as a potential etiology. Specifically, laboratory investigations have demonstrated an immunosuppressive effect of androgens [17–19], and gender-related genetic polymorphisms have been observed with respect to human proinflammatory proteins [20].

Evidence of a possible immune-related sexual dimorphism with respect to outcomes following blood product transfusion has been reported by our group and others. Specifically, transfusion-related acute lung injury appears both more frequent and severe following transfusion of female blood as compared to male blood [21, 22]. Furthermore, we observed both decreased survival and more aggressive tumor behavior for male mice, as compared to female mice, inoculated with pancreatic cancer cells following transfusion of female blood [23, 24]. Such differences may involve alloimmunization [22, 25] or even microchimerism [26] of multiparous female donors.

We have investigated the change in hematocrit (ΔHct) following pRBCs transfusion as a clinically relevant potential surrogate marker of posttransfusion hemolysis. Specifically, based upon the associations between pRBCs storage time, accumulation of inflammatory mediators [27–29], and decreased erythrocyte longevity [30], we demonstrated a significant inverse relationship between pRBCs storage time and ΔHct following transfusion [31]. The objective of the current investigation was to determine the relationship between both donor and recipient gender and ΔHct following pRBCs transfusion of critically ill surgical patients. Based upon the aforementioned observations, we hypothesized that both female donor gender and female recipient gender would be associated with a decreased ΔHct.

## 2. Material and Methods

This study was a cross-sectional analysis of all leukocyte-reduced, pRBCs transfusions administered to critically ill patients from June 2006 through June 2009 at a 20-bed academic surgical ICU. Only single-unit transfusions that occurred while the patient was in the ICU were included; multiple unit transfusions as well as transfusions administered at other locations (e.g., operating room and angiography suite) were excluded.

The independent variables of interest were both donor and recipient gender. For the purposes of this analysis, gender refers to sex chromosomal status: XX for female and XY for male. The outcome variable was the ΔHct following pRBCs transfusion (% points, continuous). ΔHct was calculated as the difference between the first posttransfusion value (Hct_post_) and the last pretransfusion value (Hct_pre_). The time from Hct_pre_ determination to the blood transfusion (T_pre_, hours), as well as the time from the blood transfusion to Hct_post_ determination (T_post_, hours) were also abstracted. In order to access for a potential effect of fluid equilibration over time, we also abstracted the second hematocrit determination following pRBCs transfusion (Hct_post2_, % points, continuous). This data point was used to calculate ΔHct_2_ (% points, continuous), defined as the difference between Hct_post2_and Hct_pre_. Transfusion of additional blood products (e.g., fresh frozen plasma, platelets, and cryoprecipitate) along with the pRBCs transfusion was also recorded.

 The following covariates were hypothesized *a priori* to potentially confound the relationship between gender and ΔHct: recipient age (years), recipient admission diagnosis (trauma versus other), the indication for transfusion (hemorrhage versus ICU anemia, the former being identified by the terms “bleeding” or “hemorrhage” used in the medical record within 48 hours of the transfusion), recipient admission weight (kg), recipient admission body mass index (BMI, kg/m^2^), recipient 24 hour fluid balance (L), time from surgical ICU admission to transfusion (days), Hct_pre_ (%), and the age of transfused blood. Recipient ICU LOS (days) as well as mortality was also abstracted.

All statistical analyses were performed using SAS version 9.2 (SAS Inc., Carey, NC, USA). The alpha error level was set at 0.05, with *P* < 0.05 being considered significant statistically, unless indicated otherwise. Continuous data are expressed as mean, range; categorical data are expressed as number, %. Correlations between predictor variables and ΔHct were assessed using Pearson's correlation coefficient (*r* ^2^). The means of two continuous variables were compared using the student's *t*-test. The means of >2 groups were compared using ANOVA (*F* statistic); individual group differences were then compared using Tukey's test in the case of overall significance. It the case of multiple comparisons Bonferroni-corrected *P*-values were used and calculated as 0.05/*k*, where *k* is equal to the number of groups analyzed. Differences in proportions of categorical variables were compared using the chi-square test, except when expected cell counts were <10, in which case Fischer's exact test was used. Multivariable linear regression (sum of squares) was used to access for the independent contribution of gender to the variability in ΔHct. Variables correlated with either recipient gender or ΔHct (or both) at the *P* ≤ 0.10 level by univariate analysis were entered into the model using a forward selection method. Overall model significance was calculated using the *F* statistic, with the significance of individual variables calculated using the *t* statistic. This study was approved by the Colorado Multiple Institutional Review Board (Protocol no.10-0028).

## 3. Results

A total of 1,011 units of pRBCs were administered during the study period. Of these, 594 (56.9%) units were given to 342 patients as single-unit transfusions. Complete data were available for 575 (96.8%) units, which served as the final sample size. Sample demographics are shown in Table 1. The vast majority of pRBCs transfusions were given for ICU anemia (91.4%), well into the recipient's ICU course (mean time from ICU admission to transfusion 13.7 days), and without additional blood products (95.1%). The mean Hct_pre_ was 21.5 (range 8.2–32). The mean pRBCs storage time was 29.5 days, and 94.3% of pRBCs units were ≥14 days old. Donor gender was distributed equally among male (49.9%) and female (50.1%) donors.

Correlations between independent variables and ΔHct are listed in Table 2. Recipient female gender (*r* ^2^ = 0.23, *P* < 0.01) and Hct_pre_ (*r* ^2^ = −0.27, *P* < 0.01) were correlated most strongly with ΔHct. Additional significant correlations [in order of decreasing magnitude (|*r* ^2^|)] were as follows: increasing recipient admission weight was associated with a decreased ΔHct (*r* ^2^ = −0.15, *P* < 0.001), transfusion for ICU anemia as opposed to hemorrhage was associated with a decreased ΔHct (*r* ^2^ = 0.13, *P* = 0.003), increasing age was associated with an increased ΔHct (*r* ^2^ = 0.10, *P* = 0.02), increasing 24 hour fluid balance was associated with a decreased ΔHct (*r* ^2^ = 0.10, *P* = 0.02), and increasing T_pre_ was associated with increased ΔHct (*r* ^2^ = 0.09, *P* < 0.04). Admission diagnosis, recipient admission BMI, time from ICU admission to transfusion, T_post_, the age of blood, donor gender, and transfusion of additional blood products with the pRBCs unit were not correlated significantly with ΔHct.

Table 3 lists sample characteristics stratified by recipient gender. As compared to female recipients, male recipients were significantly younger (*P* < 0.01), more likely to have an admission diagnosis of trauma (*P* < 0.01), more likely to be transfused for an indication of ICU anemia (*P* < 0.01), had a higher BMI (*P* < 0.01), and received older blood (*P* < 0.01).

By univariate analysis, ΔHct was significantly greater for female recipients, as compared to male recipients (3.81% versus 2.82%, resp., *P* < 0.01). However, donor gender did not impact ΔHct, which was 3.02% following receipt of female blood versus 3.23% following receipt of male blood (*P* = 0.21). In order to assess for any possible effect of fluid equilibration over time, these analyses were repeated using ΔHct_2  _instead of ΔHct. The mean ΔHct_2_ was 2.97% (range −12.3, 15.8) and the mean T_post2_ was 14.1 hours (range 1–92). ΔHct_2  _remained significantly higher for female as compared to male recipients (3.71% versus 2.63%, resp., *P* < 0.01). Furthermore, donor gender was not associated with ΔHct_2   _(2.91% following receipt of female blood versus 3.03% following receipt of male blood, *P* = 0.55).

**Figure 1:**
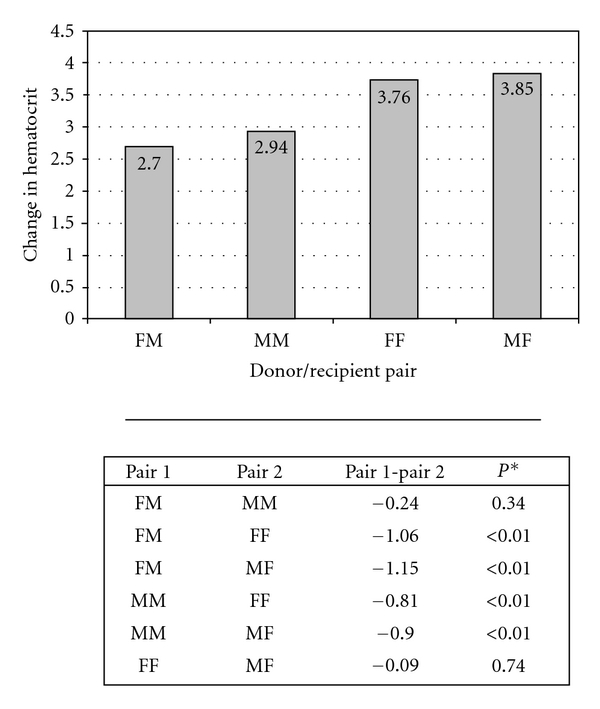
Change in Hematocrit as a function of donor-recipient gender pair. Donor-recipient pairs: FM, female donor male recipient; MM, male donor male recipient; FF, female donor female recipient; MF male donor female recipient. Pair 1 − Pair 2 = ΔHct for pair 1 − ΔHct for pair 2. P*, Bonferroni-corrected *P* value = 0.05/4 groups = 0.0125. All comparisons in which the recipient genders were discordant were significant statistically. By contrast, comparisons in which the recipient genders were identical were not significant statistically.

The relationship between both donor and recipient gender and ΔHct was explored further by stratifying the sample into the 4 donor-recipient gender pairs listed in Table 4: Male donor male recipient (MM), female donor male recipient (FM), male donor female recipient (MF), and female donor female recipient (FF). ΔHct for each of these pairs is listed in Table 4 and depicted in Figure 1. Although significant differences in ΔHct existed between these 4 groups (ANOVA DF = 3, *F* = 10.78, *P* < 0.01), analysis of differences between individual pairs revealed only those with disparate recipient genders to be significant. By contrast, ΔHct was not significantly different when comparing pairs with disparate donor genders and identical recipient genders. Differences in ΔHct as well as adjusted *P*-values for individual pair comparisons are listed in Figure 1.

Results from multivariable logistic regression analysis are shown in Table 5. The following variables were added to the model in addition to recipient gender based on their association with either recipient gender or ΔHct (or both) by univariate analysis: recipient age, admission diagnosis, indication for transfusion, Hct_pre_, recipient admission weight, T_pre_, age of blood, and recipient 24 hour fluid balance. Of note, recipient BMI was not included in the model based on a high degree of colinearity with recipient weight (*r* ^2^ = 0.90, *P* < 0.01). After controlling for these covariates, recipient gender remained significantly associated with ΔHct (*t* = 4.32, *P* < 0.01) (model DF-9, *F*-12.57, *P* < 0.01). In additional to recipient gender, patient age, Hct_pre_, recipient admission weight, and T_pre_ were also associated independently with ΔHct.

## 4. Discussion

 In this cross-sectional analysis of anemic, critically ill surgical patients, we observed an independent association between recipient gender and ΔHct following pRBCs transfusion. By contrast, a relationship between donor gender and ΔHct was not observed. Specifically, ΔHct was higher for female, as compared to male recipients, irrespective of donor gender.

Demonstration of sexual dimorphism with respect to recipient gender in this retrospective analysis raises the issues of both confounding and causality. In terms of the former, several covariates may confound the relationship between recipient gender and ΔHct. Chronologic factors, such as T_pre_ and T_post_, would appear to influence ΔHct but were not associated strongly with either recipient gender or ΔHct in this analysis, nor did they significantly alter the observed relationship between these two variables in multivariable analysis. Furthermore, because pRBCs transfusion in effect entails adding a relatively fixed volume and concentration of erythrocytes to a variable recipient volume of distribution and Hct, variables such as Hct_pre_, recipient weight, 24 hour fluid balance, and recipient BMI are of relevance. Indeed, each of these variables was correlated with ΔHct but did not explain the association between recipient gender and ΔHct. Finally, male and female recipients may have differed with respect to the condition of the donor erythrocytes themselves. Specifically, we recently observed that older blood is less effective in raising the Hct [31]. This possibility was addressed by abstracting the age of the transfused pRBCs unit. However, although females tended to receive newer blood than males (27.7 days versus 30.4 days, *P* < 0.01), this relatively small degree of difference in storage time was not found to significantly alter ΔHct in our previous work [31]. Furthermore, adding the age of blood to the regression model in this analysis did not eliminate the observed association between recipient gender and ΔHct. Thus, to the best of our ability to control for such confounding variables, female recipients were noted to raise their Hct by a greater amount than their male counterparts by a mechanism independent of either chronologic or dilutional phenomena.

The immunomodulatory properties of pRBCs transfusions involve both donor and recipient qualities. Previous work has implicated transfusion of female blood in both antibody-mediated acute lung injury [22] and cancer progression [23, 24] and invoked alloimmunization of multiparous women as one possible etiology. Similarly, androgens have been shown to possess immunosuppressive properties [19]. In this study, however, we did not observe a relationship between donor gender and ΔHct. This finding suggests that any gender-related difference in passenger immunologic components (e.g., pro-inflammatory cytokines, antibodies) did not appreciably result in hemolysis of recipient (or transfused) erythrocytes as reflected by the first two posttransfusion hematocrits. By contrast, recipient gender appeared to drive the sexual dimorphism in ΔHct, with female recipients demonstrating a significantly greater ΔHct as compared to male recipients.

We hypothesized originally a smaller ΔHct for female, as compared to male recipients based upon possible immune or sex hormone-mediated posttransfusion hemolysis. However, the opposite effect was observed. One possible explanation for this finding involves immunologic tolerance via microchimerism. Microchimerism entails proliferation of a relatively small (<5%) population of cells (e.g., leukocytes) from a distinct zygote and as a result of mixing of blood between the two organisms. Examples of circumstances leading to microchimerism include solid organ transplantation, pregnancy, and blood transfusion itself. One hypothesis for the observed findings thus involves a lesser degree of immune-mediated hemolysis of transfused pRBCs in female, as compared to male recipients, as a result of microchimerism-mediated immune tolerance. Although testing of this hypothesis is beyond the scope of our dataset, microchimerism and its relationship to immune tolerance represent an important development in the field of transfusion that may help to elucidate the sexual dimorphism observed in various outcomes of critically ill patients. Beyond microchimerism, immunologically relevant gender differences in sex hormone expression may contribute to the observed sexual dimorphism in ΔHct. Continued research is necessary to elucidate and potentially manipulate the observed gender-related disparity in the hopes of ultimately decreasing both patient exposure to and morbidity of allogeneic blood product transfusion.

Out study is limited by a retrospective design. We were unable to capture additional variables that may have influenced ΔHct irrespective of recipient gender such as donor and recipient sex hormone concentrations, menopausal status, and obstetric and transfusion histories. Markers of hemolysis, such as haptoglobin, bilirubin, lactate dehydrogenase, and iron, as well as the presence of hemoglobinuria, were not measured routinely. Demonstration of an association between these markers and ΔHct would provide further evidence for the hemolysis hypothesis and is the focus of a current prospective study in our surgical ICU. Study strengths include a large and relatively uniform sample of critically ill surgical patients, rigorous collection of chronologic and demographic variables, and a low percentage of missing data.

## 5. Conclusions

 Among this cohort of critically ill surgical patients, we observed a significant association between recipient gender and ΔHct following pRBCs transfusion, such that female recipients raised their hematocrit more than male recipients. This relationship was independent of either chronologic or anthropomorphic factors, differences in the age of transfused blood, and the gender of the donor. No relationship was observed between donor gender and ΔHct in any analysis. Additional factors that were associated independently with ΔHct included recipient age, recipient admission weight, Hct_pre_, and T_pre_. The etiology of the observed sexual dimorphism is unknown but may involve gender-related differences in immune-mediated hemolysis of transfused erythrocytes; additional research at our institution is focused on exploring this hypothesis.

## Figures and Tables

**Table 1:** Sample demographics.

Variable (*n* = 575)	Mean/*N*	Range/%
Recipient characteristics		
Age (years)	49.3	18–93
Male gender	398	68.7%
Admission diagnosis of trauma	384	66.3%
Transfusion indication of ICU anemia	529	91.4%
Admission weight (kg)	82.2	40.8–185.0
Admission BMI (kg/m^2^)	27.9	16.8–66.3
24 Hour fluid balance (L)	2.1	−5.6–55.9

Transfusion data		
Time from ICU admission to	13.7	1–71
transfusion (days)		
Hct_pre_ (% points)	21.5	10.2–32
T_pre _ (hours)	3.3	1–7
T_post_ (hours)	4.6	1–6
pRBCs storage time (days)	29.5	7–42
Donor male gender	289	49.9%
Additional blood products	28	4.9%

Outcomes		
ΔHct (% points)	3.1	−7.0–16.9
ICU LOS (days)	27.4	1–111
Recipient mortality	119	20.7%

ICU, intensive care unit; BMI, body mass index; pRBCs, packed red blood cells; LOS, length of stay.

**Table 2:** Univariate correlations with ΔHct.

Variable	*r* ^2^	*P*
Recipient characteristics		
Age (years)	0.10	0.02
Male gender	−0.23	<0.01
Admission diagnosis of trauma	−0.01	0.74
Transfusion indication of ICU anemia	0.13	<0.01
Admission weight (kg)	−0.15	<0.01
Admission BMI (kg/m^2^)	−0.02	0.63
24 Hour fluid balance (L)	−0.10	0.02

Transfusion data		
Time from ICU admission to transfusion (days)	−0.04	0.35
Hct_pre_ (% points)	−0.27	<0.01
T_pre_ (hours)	0.09	0.04
T_post_ (hours)	0.05	0.27
pRBCs storage time (days)	−0.01	0.68
Donor male gender	0.05	0.21
Additional blood products	−0.06	0.13

*r* ^2^, Pearson's correlation coefficient; ICU, intensive care unit; BMI, body mass index; pRBCs, packed red blood cells.

**Table 3:** Sample characteristics stratified by recipient gender.

Variable	Male Recipient (*n* = 398)	Female recipient (*n* = 177)	*P*
Recipient characteristics			
Age (years)	48.0	52.5	<0.01
Admission diagnosis of trauma	296 (74.8%)	87 (48.6%)	<0.01
Transfusion indication of ICU anemia	351 (88.6%)	172 (97.2%)	<0.01
Admission weight (kg)	82.7	81.2	0.44
Admission BMI (kg/m^2^)	30.9	26.6	<0.01
24 Hour fluid balance (L)	2.0	2.1	0.73

Transfusion data			
Time from ICU admission to transfusion (days)	14.1	12.8	0.22
Hct_pre_ (% points)	21.6	21.3	0.13
T_pre_ (hours)	3.2	3.3	0.73
T_post_ (hours)	4.5	4.7	0.69
pRBCs storage time (days)	30.4	27.7	<0.01
Donor male gender	197 (49.8%)	92 (51.4%)	0.71
Additional blood products	16 (4.0%)	12 (6.7%)	0.17

ICU, intensive care unit; BMI, body mass index; pRBCs, packed red blood cells.

**Table 4:** Change in hematocrit following transfusion stratified by donor-recipient gender pairs.

Donor		Recipient		*N*	ΔHct
Male	Female	Male	Female		
•		•		197 (34.0%)	2.94
	•	•		201 (34.7%)	2.48
•			•	92 (15.9%)	3.85
	•		•	89 (15.4%)	3.76

**Table 5:** Multivariable linear regression.

Variable	Parameter estimate	Standard error	*P*
Recipient age (years)	0.016	0.005	<0.01
Admission diagnosis of trauma	0.240	0.182	0.19
Transfusion indication of ICU Anemia	0.434	0.290	0.13
Hct_pre _(% points)	−0.269	0.036	<0.01
Recipient admission weight (kg)	−0.013	0.004	<0.01
T_pre_ (hours)	0.073	0.027	<0.01
pRBCs storage time (days)	0.001	0.009	0.95
24 Hour fluid balance (L)	−0.001	0.001	0.08
Recipient female gender	0.793	0.183	<0.01

ICU, intensive care unit; pRBCs, packed red blood cells; model DF = 9, *F* = 12.57, *P* < 0.01.
